# Supplementary material for: SST-ResNet: A Sequence and Structure Information Integration Model for Protein Property Prediction
Source: Int J Mol Sci. 2025 Mar 19;26(6):2783. doi: 10.3390/ijms26062783 (PMC11942962; doi:10.3390/ijms26062783)
Supplement: Supplementary file 1 [file ijms-26-02783-s001.zip › ijms-3522311-supplementary.pdf]

Supplementary Information

SST-ResNet: A sequence and structure information integration model for protein property prediction

Guowei Zhou [1,2†], Yanpeng Zhao [2,3†], Song He [2,*] and Xiaochen Bo [1,2*]

1 Academy of Medical Engineering and Translational Medicine, Tianjin University, Tianjin 300072, China; 2022235039@tju.edu.cn

2 Academy of Military Medical Sciences, Beijing 100850, China; zyp182531903@163.com

3 School of Medicine, Shanghai University, Shanghai 200444, China

* Correspondence: hes1224@163.com; boxiaoc@163.com

† These authors contributed equally to this work.

Supplementary Table S1. The training details of SST-ResNet.

Hyperparameter	Description	Range
batch size	Input batch size	{16,32,64,128}
lr	Initial learning rate for SST-ResNet	{1e-4,5e-5,3e-5,1e-5}
dropout	Dropout ratio for the SST-ResNet	{0,0.2}
n_layer	Number of layers in SST-ResNet	{2,3,4,6,8,10,12}
hidden_size	Size of layers in SST-ResNet	{128,256,512,1024}
kernal size	Kernal size of layers in SST-ResNet	{3,5,7,9,11,13,15}
activation	Nonlinear activation function in SST-ResNet	{ReLu,GeLu}

Supplementary Table S2. Ablation experiment details of SST-ResNet - Fmax.

Task	SST-ResNet	SR-kernal3	SR-kernal5	SR-kernal7	ProSST
EC	$0.858_{\pm 0.001}$	$0.82_{\pm 0.003}$	$0.835_{\pm 0.000}$	$0.837_{\pm 0.004}$	$0.376_{\pm 0.003}$
GO-MF	$0.613_{\pm 0.002}$	$0.582_{\pm 0.002}$	$0.597_{\pm 0.002}$	$0.601_{\pm 0.002}$	$0.281_{\pm 0.001}$
GO-BP	$0.425_{\pm 0.007}$	$0.396_{\pm 0.009}$	$0.413_{\pm 0.003}$	$0.427_{\pm 0.003}$	$0.308_{\pm 0.001}$
GO-CC	$0.463_{\pm 0.02}$	$0.427_{\pm 0.012}$	$0.436_{\pm 0.004}$	$0.451_{\pm 0.011}$	$0.399_{\pm 0.001}$

Supplementary Table S3. Ablation experiment details of SST-ResNet - AUPR.

Task	SST-ResNet	SR-kernal3	SR-kernal5	SR-kernal7	ProSST
EC	$0.81_{\pm 0.001}$	$0.754_{\pm 0.006}$	$0.781_{\pm 0.003}$	$0.789_{\pm 0.006}$	$0.251_{\pm 0.002}$
GO-MF	$0.521_{\pm 0.001}$	$0.494_{\pm 0.006}$	$0.51_{\pm 0.005}$	$0.513_{\pm 0.01}$	$0.151_{\pm 0.001}$
GO-BP	$0.249_{\pm 0.004}$	$0.228_{\pm 0.001}$	$0.239_{\pm 0.002}$	$0.246_{\pm 0.001}$	$0.093_{\pm 0.000}$
GO-CC	$0.271_{\pm 0.01}$	$0.254_{\pm 0.003}$	$0.256_{\pm 0.002}$	$0.268_{\pm 0.005}$	$0.12_{\pm 0.001}$

Supplementary Table S4. The Investigation of SST-ResNet representation details.

Hyperparameter	Description	Settings
random seed	The random seed numbers of PCA and UMAP.	42
pca' s n_components	Number of principal components to retain.	50
umap' s n_neighbors	Number of neighboring points used to approximate local data structure.	15
umap' s min_dist	Minimum distance between points in the low-dimensional embedding	0.01
DBSCAN' s eps	Maximum distance between two samples for them to be considered as in the same neighborhood.	0.4
DBSCAN' s min_samples	Minimum number of samples required to form a dense region.	2
